# Patterns of Acute Gamma‐Hydroxybutyrate Harms Requiring Ambulance Attendance: Should Greater Focus Be on Regional Areas?

**DOI:** 10.1111/dar.14086

**Published:** 2025-05-28

**Authors:** Naomi Beard, James Wilson, Bosco C. Rowland, Ziad Nehme, Dan I. Lubman, Rowan P. Ogeil

**Affiliations:** ^1^ Turning Point, Eastern Health Melbourne Australia; ^2^ Barwon Health Geelong Australia; ^3^ Eastern Health Clinical School and Monash Addiction Research Centre Monash University Melbourne Australia; ^4^ Centre for Research and Evaluation, Ambulance Victoria Melbourne Australia; ^5^ Department of Epidemiology and Preventative Medicine Monash University Melbourne Australia; ^6^ Department of Paramedicine Monash University Melbourne Australia

**Keywords:** ambulance attendances, GHB, paramedics, pre‐hospital

## Abstract

**Introduction:**

Gamma‐hydroxybutyrate (GHB) use and attributable harms have been increasing in Europe and Australia. However, there are limited population surveillance tools available to map and track acute GHB‐related harms, particularly outside metropolitan areas. The present study examined GHB‐related ambulance attendances from January 2015 to March 2024 across the state of Victoria, and in Greater Geelong, the region associated with the highest number of attendances outside the state capital.

**Methods:**

Retrospective analysis of all GHB‐related ambulance attendances between 1 January 2015 and 31 March 2024 from the Victorian arm of the National Ambulance Surveillance System. Descriptives and time series analyses were used to present demographic and spatio‐temporal patterns.

**Results:**

There were 16,971 ambulance attendances for GHB during the study period. A sinusoidal trend was apparent in the statewide data, suggesting a seasonal factor to GHB‐related attendances, with greater numbers occurring during quarter four of each year. Whilst a seasonal effect was also apparent in Greater Geelong, increases in attendances have been consistent since quarter four of 2021 (between 7% and 34%). The magnitude of these increases was not observed in other regional areas.

**Discussion and Conclusions:**

Acute GHB‐related harms have increased in Victoria over time, in addition to a seasonal effect being apparent that coincided with summer in the Southern Hemisphere. Our findings support recent media reports from emergency department workers in the region of Greater Geelong that GHB harms have risen. This study demonstrates the value of using ambulance surveillance data to assess pre‐hospital harms resulting from GHB use.

## 1 Introduction

Gamma‐hydroxybutyrate (GHB) is a central nervous system depressant traditionally associated with the club, dance and Chemsex scenes [1]. Rises in acute GHB‐related harms have been reported in Australia [2, 3] and Europe [1, 4] and are cause for concern given the severity of reported harms which include ambulance attendance, hospitalisation and overdose deaths [4, 5, 6]. GHB harms are disproportionally spread across the population with higher rates in younger people (< 30 years) [7, 8], lower socioeconomic and/or regional areas [6], and gender diverse populations across Australia [9].

The number and severity of harms experienced following GHB use have been increasing, including death rates assessed in the National Coronial Information System since 2016 [3]. Morbidity related to GHB harms has also increased between 2012 and 2019, with a 147% increase in the rate of GHB‐related ambulance attendances observed in the state of Victoria [2], a trend that continued into 2020 (*n* = 2622 attendances) with more than half involving an acute overdose (56%) or altered consciousness (45%) [8]. In this state‐wide study, attendances in regional Victoria were 31% more likely to present with more severe outcomes compared with attendances in metropolitan areas [8], consistent with a European study conducted in the Netherlands and Belgium that found that people using GHB and living in regional areas had experienced a GHB‐related coma more often than those in cities [6]. Despite the acknowledgement of increased GHB‐related harms in regional settings, there is a general paucity of knowledge and understanding of the patterns of GHB harms occurring in regional settings, and few population‐based datasets that can track these over time.

Most literature examining GHB use and related harms has methodological limitations, including a reliance on survey methods or interviews which may be prone to recall and social desirability biases, and/or data collection protocols focussed on metropolitan‐based participants [9, 10]. These limitations could be overcome by use of acute care clinical records, which contain information related to specific drug types, although they are not routinely examined. Ambulance records, in particular, provide a unique opportunity to examine acute harms attributable to GHB use [7, 8, 11], and unlike emergency department or hospital records, provide state‐wide coverage allowing for an examination of regional differences [2, 8]. The present paper utilised a novel surveillance system which includes coded ambulance data to investigate GHB‐related harms in Victoria [12], building on previous studies in Europe [7] and Australia [2, 8, 11] that have demonstrated the ability of ambulance records to provide timely identification of GHB‐related harms.

## 2 Methods

### 2.1 Data Source

The National Ambulance Surveillance System (NASS) is an internationally unique public health surveillance system filtered for capturing alcohol and other drug, mental health and self‐harm related events ambulance attendances [12, 13]. The NASS comprises electronic patient care records attended by jurisdictional ambulance services. Attendances are given a unique patient identifier, which includes demographic, geographic and clinical details. Data are imported to a custom‐built coding database, where the records are examined by trained research assistants following validated protocols. GHB‐related attendances were coded when cases met the following criterion: “Is it reasonable to attribute the immediate or recent use of GHB as a contributing reason for the ambulance attendance?” Further details on the NASS and on variables included are described in the following methods papers [12, 13].

The Eastern Health Research Ethics Committee provided ethics approval (#E122/0809). The study follows the “Strengthening the Reporting of Observational Studies in Epidemiology” (STROBE) guidelines.

### 2.2 Study Location

We investigated characteristics of spatio‐temporal patterns in ambulance attendances related to GHB, focusing on the regional Victorian Local Government Area (LGA) of Greater Geelong, given recent media reports highlighting increased presentations to emergency departments for GHB [14]. Victoria is comprised of 79 LGAs, 31 of which are in metropolitan Melbourne and 48 in regional Victoria. Geelong is the 2nd most populous city in the state of Victoria after the state capital Melbourne, and the Greater Geelong LGA had 271,057 residents according to the 2021 Census. We included all GHB‐related attendances between January 2015 and March 2024 (noting that Ambulance Victoria undertook industrial action on 18 March 2024 until 23 September 2024, resulting in reduced reporting of attendance numbers throughout this period) across Victoria, with a sub‐analysis focusing on the LGA of Greater Geelong. Case ascertainment was determined from an examination of paramedic clinical case notes, where clinical observations, demographic information and contextual factors are recorded [12, 13].

### 2.3 Data Analysis

Descriptive analyses were used to characterise attendance demographics. Categorical variables including gender, geography, transportation to hospital and police co‐attendances are reported as numbers and proportions. Age is reported as a median, and crude rates (per 100,000 population) were calculated separately using the 2021 Census population data retrieved from the Australian Bureau of Statistics. Non‐binary genders were removed from analysis as these are not well captured and are underrepresented in the NASS, accounting for 0.1% (*n* = 22) of GHB‐related attendances over the nine‐year study period. Time‐series analyses for all of Victoria and Greater Geelong were aggregated into quarters and plotted by total number of GHB attendances over time. Percentage changes in the total number of GHB‐related ambulance attendances were calculated by subtracting the number of attendances (*v*2) from the previous quarter (*v*1), then dividing by the number of the previous quarter (*v*1) and multiplying the value by 100 ($\frac{v2-v1}{v1}$ × 100). Percentage changes were plotted quarter‐on‐quarter for all of Victoria and Greater Geelong. Positive changes indicate a percentage increase compared to the previous quarter and represents an increase in the overall number of cases. The commencement of the COVID‐19 pandemic in quarter one 2020 is acknowledged with a graphical marker. All analyses were performed in Stata version 15.0.

## 3 Results

A total of 16,971 GHB‐related ambulance attendances between 1 January 2015 and 29 March 2024 were identified (Table 1). Most were for young males (55%), with a median age of 28 years. Polydrug use involving GHB accounted for 50% of attendances across Victoria. Between 2015 and 2024, the LGA of Melbourne accounted for the largest proportion of GHB attendances (*n* = 2867, 17%) across the state, and the LGA of Greater Geelong accounted for one‐third (31%) (*n* = 676) of all GHB attendances in regional Victoria (*n* = 2209). Between 2015 and February 2024, Greater Geelong was ranked fourth out of all LGAs in the state by total number of GHB‐related ambulance attendances (*n* = 676), the only regional LGA ranked inside the top 20, with the next regional LGA, Latrobe, ranked 27th.

**TABLE 1:** Demographic characteristics of GHB‐related ambulance attendances by geography, January 2015 to March 2024, Victoria, Australia.

Characteristic	Sub‐category	*n* (%)
Victoria		
Attendances (*n*)		16,971
Crude rate of attendance (per 100,000)^a^		261.0
Gender	Male	9334 (55.0)
	Female	7637 (45.0)
Age, years (median)		28
Geography	Metropolitan Melbourne	14,762 (87.0)
	Regional Victoria	2209 (13.0)
Top 5 local government areas (statewide)	Melbourne	2867 (16.9)
	Port Phillip	834 (4.9)
	Casey	810 (4.8)
	Greater Geelong	676 (4.0)
	Stonnington	646 (3.8)
GHB polydrug use^b^	Yes	8511 (50.2)
Transport to hospital	Yes	14,147 (83.4)
Police co‐attendance	Yes	7687 (45.3)
Metropolitan Melbourne		
Attendances (*n*)		14,762
Crude rate of attendance (per 100,000)^a^		296.9
Gender	Male	8163 (55.3)
	Female	6599 (44.7)
Age, years (median)		28
Top 5 local government areas (metropolitan Melbourne)	Melbourne	2867 (19.6)
	Port Phillip	834 (5.7)
	Casey	810 (5.6)
	Frankston	649 (4.4)
	Stonnington	646 (4.4)
GHB polydrug use^b^	Yes	7305 (50.0)
Transport to hospital	Yes	12,307 (83.4)
Police co‐attendance	Yes	6666 (45.2)
Regional victoria		
Attendances (*n*)		2209
Crude rate of attendance (per 100,000)^a^		139.3
Gender	Male	1171 (53.0)
	Female	1038 (47.0)
Age (median)		28
Top 5 local government areas (regional victoria)	Greater Geelong	676 (30.6)
	Latrobe	212 (9.6)
	Greater Bendigo	208 (9.4)
	Ballarat	159 (7.2)
	Baw Baw	105 (4.8)
GHB polydrug use^b^	Yes	1131 (51.2)
Transport to hospital	Yes	1840 (83.3)
Police co‐attendance	Yes	1021 (46.2)

^a^ Crude rates calculated using the following denominators: Victoria (6,503,491), Metropolitan Melbourne (4,917,750), Regional Victoria (1,585,738).

^b^ GHB polydrug use refers to any attendance involving GHB and one or more of the following substances: alcohol, amphetamines, cannabis, cocaine, ecstasy, heroin, ketamine, other illicit substance, anticonvulsant, antidepressant, antipsychotic, benzodiazepine, oxycodone, codeine, other opioid analgesic, opioid substitution therapy, non‐opioid analgesics, pharmaceutical stimulant, other pharmaceutical, other substance and unknown substance.

**FIGURE 1:**
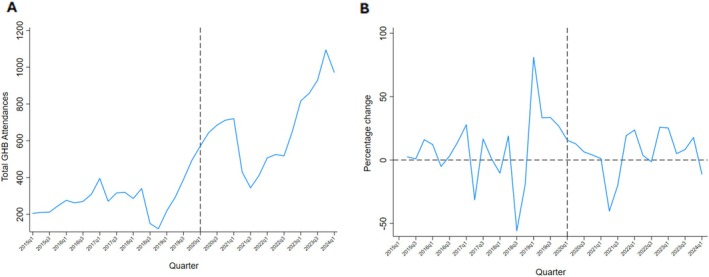
Number and percentage change of GHB‐related ambulance attendances by quarter of the year, Victoria January 2015–March 2024.

We plotted the number of GHB‐related attendances across Victoria between quarter one (q1) 2015 and quarter one (q1) 2024 (Figure 1a). A seasonal effect was observed, with the plotted line indicating an increase in GHB‐related attendances. The sinusoidal pattern suggests that more attendances occurred during quarter four of each year, coinciding with the Australian summer and subsequent music festival season. Figure 1b indicates the percentage change for each quarter. Prior to 2018q2, the seasonal percentage change per quarter ranged between +28% and −31%. Prior to COVID‐19 (2020q1), there was an 81% increase in GHB‐related attendances compared to the previous quarter. This gradually declined over the COVID‐19 lockdown periods in 2020 and 2021. Since 2022, there has been mostly increasing seasonal changes per quarter, ranging between +5% and +25%.

**FIGURE 2:**
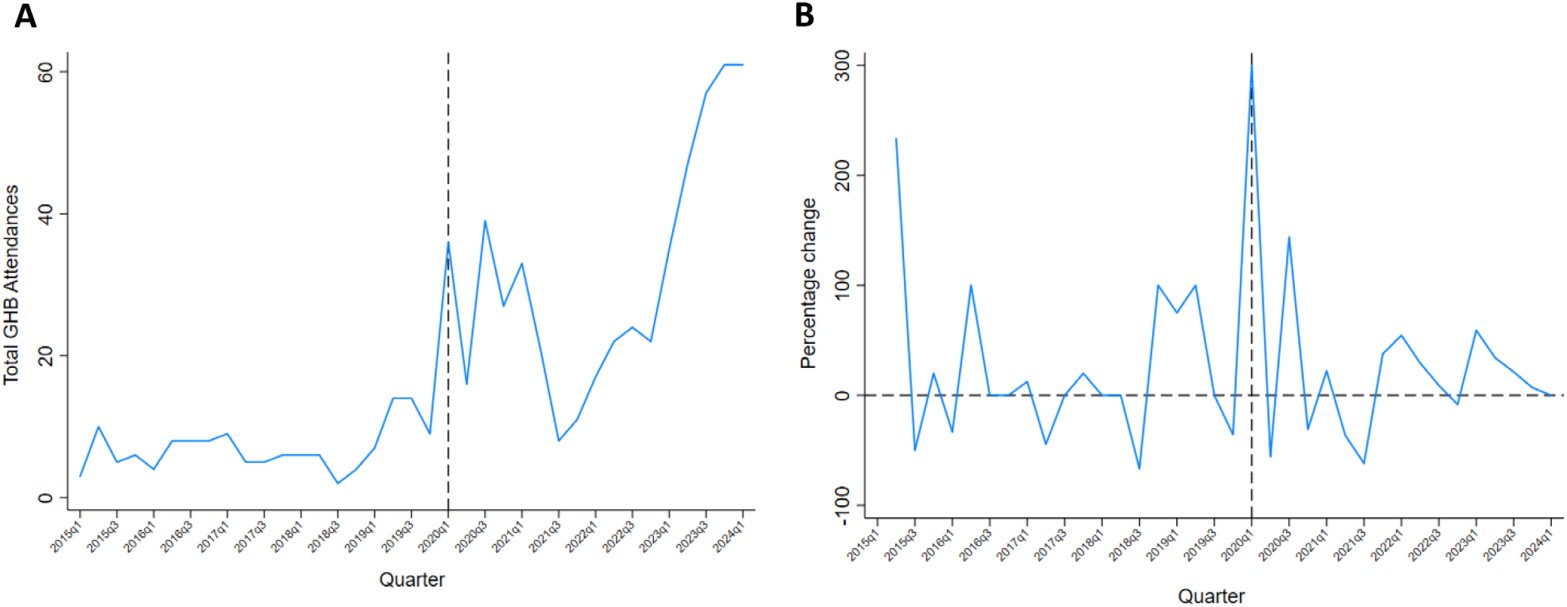
Number and percentage change of GHB‐related ambulance attendances by quarter of the year, Greater Geelong January 2015–March 2024.

Figure 2a shows the plotted number of GHB‐related attendances between 2015q1 and 2024q1 for the LGA of Greater Geelong. A seasonal effect was observed in Greater Geelong, with an increase in the number of attendances. Figure 2b indicates the percentage change for each quarter. Before COVID‐19 (2020q1), the percentage change for each quarter ranged between +100% and −44%. Just prior to COVID‐19 (2020q1), there was a 300% increase in GHB‐related attendances. This declined by 55% the next quarter and then increased by 144% in the following quarter. After 2021q4, there have been mostly increasing seasonal changes per quarter, ranging between +7% and +34%. The magnitude of these increases has not been observed in other regional Victorian LGAs.

## 4 Discussion

This study examined spatio‐temporal patterns of GHB‐related harms in Victoria, Australia using data from an ambulance surveillance system. Our results highlight the utility of this source to map acute‐GHB harms and demonstrate that they can be used to assess patterns at a state‐based level, as well as at a localised level. Greater Geelong was identified as a regional location of concern, with 31% of GHB attendances outside of metropolitan Melbourne occurring in this LGA.

Our findings of increased harms outside of metropolitan areas support studies in Europe [6] and local media reports from emergency department clinicians in Victoria [14] as to increases in GHB‐related harms in regional areas, with increasing ambulance attendances for GHB in 2023 compared with previous years. Seasonal changes in GHB‐related harms have previously been identified in international literature [4, 7] however, these changes vary in context to the countries in which they occur. The narrow therapeutic window coupled with the increased popularity of using GHB with other substances is a cause for concern in that current public health approaches focussed on drug literacy and harm reduction initiatives may not be reaching the populations most at risk of adverse outcomes, particularly younger people. Qualitative studies have identified major gaps in the understanding of signs of GHB overdose, the risks of unconsciousness related to GHB use, misconceptions on home remedies for reversing or preventing GHB overdose, and the circumstances deemed necessary for emergency service intervention [6, 9, 15]. Whilst overall population‐based metrics associated with GHB use may show stability of decreases, there are likely vulnerable groups who may have increased their use of GHB [1] or be using it in a riskier manner that cannot be easily identified without consideration of acute harm‐based data such as paramedic attendance.

Drug testing services are not currently available in most Australian states, despite robust domestic and international evidence demonstrating the efficacy of such services in providing timely education and support, as well as reducing community harms and the number of tainted products on the black‐market [10, 16, 17, 18, 19, 20]. Across Australia, concerns of dangerous and toxic adulterants in batches of GHB have been raised [16], underscoring the need for safeguards, such as drug checking services which can be fixed (permanent at a given site), or mobile (e.g., when run at a specific festival). Given the seasonal trends evident in our findings, one target for this is likely festivals held over the summer period. In the well‐known music festival “*Groovin the Moo*” hosted in the Australian Capital Territory (CANTest trial), information generated allowed local health services to issue informative alerts on illicit drugs [18, 19, 20]. In contrast, a UK study trialled drug checking services in the community, allowing a broader population of people to check their substances before consumption [10]. Both studies reported young men (< 35 years) as the primary users of their services and reported similar harm reduction outcomes, including alerting friends of identified contaminants, lowered, or adjusted dosage, disposal of substances, and higher caution with poly‐substance use [10, 18, 19].

The Victorian state government announced an 18‐month implementation trial of drug checking services over the 2024/25 Australian summer (including at Beyond the Valley in Geelong). The Beyond the Valley mobile service reported having tested more than 600 samples over the four‐day festival [21]. More than 70% of the 700 festivalgoers interviewed by harm reduction workers expressed that this was the first time they had engaged in an honest and judgement‐free conversation about drug and alcohol safety with a healthcare professional [21], highlighting the extent to which drug checking services offer more than just testing of substances, but vital opportunities to have conversations around reducing drug‐related harm. A fixed site is also set to open in 2025, located in an inner metropolitan Melbourne area [21], which, similar to the drug checking service in the Australian Capital Territory (CANTest), will provide coverage that extends beyond single events. Our findings suggest that other fixed site services should also be considered, particularly in the Greater Geelong region.

There is a gap in the current conversation when addressing harm reduction strategies for GHB [6, 9]. As GHB and its chemical precursors gamma butyrolactone and 1,4‐butanediol have become more popular in Australia and abroad, more drug seizures, border detections, deaths and non‐fatal overdoses have been observed, as well as the number of people seeking emergency treatment for associated harms [4, 5, 7, 8, 14, 22]. These harms have been associated with concern in regional areas in Europe [6, 7] and Australia [3, 8, 9, 14], with the rate of overdose deaths in Victoria consistent across regional and metropolitan areas [5].

### 4.1 Strengths and Limitations

NASS is an internationally unique and ongoing resource that captures acute alcohol and other drug‐related harms in pre‐hospital settings. However, a limitation is that NASS comprises data that were collected for administrative and clinical purposes, rather than for research purposes [12, 13]. Therefore, information pertaining to drug availability, where the drug was consumed, and the type of harm experienced is unavailable. Additionally, 50% of GHB attendances in our study also involved another substance (polysubstance), consistent with previous research [2, 8]; however, further investigation of this finding by specific drug combinations used is outside the scope of this study. Although non‐binary persons are a key at‐risk group for GHB‐related harms, attendances involving non‐binary persons were omitted from analyses due to underrepresentation in the NASS. The results from this study, notably the slight percentage change decreases observed in quarter one of 2024, should be interpreted with caution, acknowledging that data from 18 March 2024 (quarter one 2024) was affected by industrial action at Ambulance Victoria resulting in reduced reporting of attendances. Future studies should consider examining these factors using complementary methods to understand the possible social, political and economic reasons behind the observed increases in GHB‐related harms in specific local regions.

## 5 Conclusions

Consistent increases in GHB‐related ambulance attendances were observed across Victoria, and specifically Greater Geelong, complementing other data sources and media reports of harm linked to GHB use. Ambulance data provide the opportunity for surveillance of drug harms to localised areas, including outside of major cities. Our findings underscore the need for population‐level interventions tailored to engage younger people, as well as the need for drug checking services, with particular focus on addressing the risk faced by those outside of major cities.

## Author Contributions

Each author certifies that their contribution to this work meets the standards of the International Committee of Medical Journal Editors.

## Conflicts of Interest

Ziad Nehme is supported by a National Heart Foundation fellowship (#105690). Dan I. Lubman is supported by a NHMRC Leadership Fellowship (#1196892).

## Data Availability

The datasets generated and analysed for the current study are not publicly available due to the need to protect privacy and confidentiality. Ambulance data are provided to Turning Point under strict conditions for the storage, retention and use of the data. Researchers wishing to undertake additional analyses of the data are invited to contact Turning Point as the data custodians at info@turningpoint.org.au.
